# Investigation of Nonlinear Optical Properties of Quantum Dots Deposited onto a Sample Glass Using Time-Resolved Inline Digital Holography

**DOI:** 10.3390/jimaging8030074

**Published:** 2022-03-16

**Authors:** Andrey V. Belashov, Igor A. Shevkunov, Ekaterina P. Kolesova, Anna O. Orlova, Sergei E. Putilin, Andrei V. Veniaminov, Chau-Jern Cheng, Nikolay V. Petrov

**Affiliations:** 1 Ioffe Institute, 194021 Saint-Petersburg, Russia; belashov.andrey.93@gmail.com; 2 Faculty of Information Technology and Communication Sciences, Tampere University, 33720 Tampere, Finland; igor.shevkunov@tuni.fi; 3 Faculty of Photonics, ITMO University, 197101 Saint-Petersburg, Russia; kolesovaekaterina@itmo.ru (E.P.K.); a.o.orlova@itmo.ru (A.O.O.); avveniaminov@itmo.ru (A.V.V.); 4 Information Optics and Photonics Laboratory, Institute of Electro-Optical Engineering, National Taiwan Normal University, Taipei 11677, Taiwan; cjcheng@ntnu.edu.tw

**Keywords:** nonlinear refractive index, noncollinear degenerate phase modulation, femtosecond pulses, quantum dots, inline digital holograms, pump probe digital holography, diffraction patterns

## Abstract

We report on the application of time-resolved inline digital holography in the study of the nonlinear optical properties of quantum dots deposited onto sample glass. The Fresnel diffraction patterns of the probe pulse due to noncollinear degenerate phase modulation induced by a femtosecond pump pulse were extracted from the set of inline digital holograms and analyzed. The absolute values of the nonlinear refractive index of both the sample glass substrate and the deposited layer of quantum dots were evaluated using the proposed technique. To characterize the inhomogeneous distribution of the samples’ nonlinear optical properties, we proposed plotting an optical nonlinearity map calculated as a local standard deviation of the diffraction pattern intensities induced by noncollinear degenerate phase modulation.

## 1. Introduction

Much attention has been paid to the investigation of the optical nonlinear properties of various samples and materials [1,2] as well as to the development of high-precision techniques for the implementation of such measurements. The relevance of this problem is mostly due to a large number of novel optical materials being developed in the last decade. Many studies have also reported the development of novel optoelectronic devices based on the proposed materials [3,4,5]. One of the key material parameters highly relevant for some of these developments and responsible for nonlinear photorefractive response is the nonlinear refractive index. In recent years, many studies and reviews focused on the estimation of this parameter in some emerging materials, including nanostructures and quantum dots (QDs) [6,7,8,9,10]. These studies have shown that the nonlinear refractive indices of QDs in aggregates and nanofilms can significantly exceed (by 3–6 orders of magnitude) the nonlinear refractive indices of traditional homogeneous optical media [11,12,13,14,15,16].

Until now, several approaches to measuring the nonlinear optical properties of a material have been proposed. These methods were based on Z-scan approach [7], interferometric pump-probe configuration [17], integrated digital holography for measuring thermal lens (TL) and thermal mirror (TM) effects induced by femtosecond laser pulses [18], time-resolved digital holographic microscopy [19,20], etc. Additionally, investigations of the spatial distribution of the nonlinear refractive index of inhomogeneous samples have been reported using point-by-point scanning of the sample [21,22].

In our previous study [23], a novel time-resolved inline digital holography (TRIDH) approach was proposed for the evaluation of the nonlinear refractive index of homogeneous optical media with a constant nonlinear refractive index. The method was based on an analysis of an inline digital hologram set recorded at a relatively small distance from the object plane. A stepwise comparison of numerically simulated diffraction patterns obtained at various sets of parameters with experimentally recorded data allowed for determining the samples’ optical properties while minimizing fitting errors.

In contrast with other methods for the investigation of nonlinear optical phenomena (e.g., [20,21,24,25]), the TRIDH approach can be implemented on a lensless optical setup where the pump beam is collimated [26], and no requirement of extremely high power density of a focused lased beam is needed. It provides higher stability of the optical setup and enables noninvasive investigation of the optical materials with a low destruction threshold level.

In this work, we present an experimental validation of the method on a sample glass partially covered with several layers of QDs. The remaining part of the paper contains the following: In Section 2 and Section 3, the sample preparation and experimental setup for recording the inline digital holograms data set are described. Section 4 presents the evaluation of the glass substrate nonlinear refractive index within the area, where no QDs were deposited, similar to the process described in [23]. Section 5 and Section 6 discuss the more complex case of inhomogeneously distributed nonlinear optical properties, the evaluation of a QD nonlinear refractive index, and estimation of the general “nonlinearity map” of the sample.

## 2. QD Sample Preparation

Colloidal CdSe/ZnS QDs of the core-shell type were obtained as a result of high-temperature organo-metallic synthesis by hot injection according to the protocol described in [27]. We used CdSe/ZnS QD ensembles with average core diameters within the range of 3.5–5.5 nm. During QD synthesis, their surface was coated with trioctylphosphine oxide (TOPO) molecules, which provided the QDs with solubility in hydrophobic solvents and prevented spontaneous QD aggregation. The formation of nanoparticle layers was carried out using a KN2002 Langmuir-Blodgett trough (KSV NIMA, Sweden). Distilled water with a conductivity of 18 MΩ/cm was poured into a small bath. Afterward, the water surface between the barriers was cleaned using an air pump to achieve a water film surface tension of less than 0.01 mN/cm (which corresponds to a clean water surface). A Wilhelm sensor was used to measure the surface tension force. The substrates were vertically lowered into water that was purified from contamination. Then, the water surface was cleaned again. When the required purity degree was achieved, a colloidal solution of nanoparticles was evenly distributed onto the water surface.

It was experimentally found that complete evaporation of hexane from the water surface takes place within 15 min. After the distribution of the nanoparticles on the surface of the water, the nanoparticles were allowed to spread evenly over the surface of the water for the next 30 min. Then, the film of nanoparticles was formed by reducing the film area and by increasing the surface tension by moving the barriers. Using the movable surface barriers, the film can be compressed to the form of a monolayer with close packing of the nanoparticles, in which the area per nanoparticle corresponds to the cross-sectional area of the nanoparticle. The transfer of the film from the water surface to the substrate was carried out using vertical deposition technology. During the sample preparation, QDs were deposited only on half of the glass substrates to provide better experimental validation of the TRIDH method.

## 3. Experimental Setup

**Figure 1:**
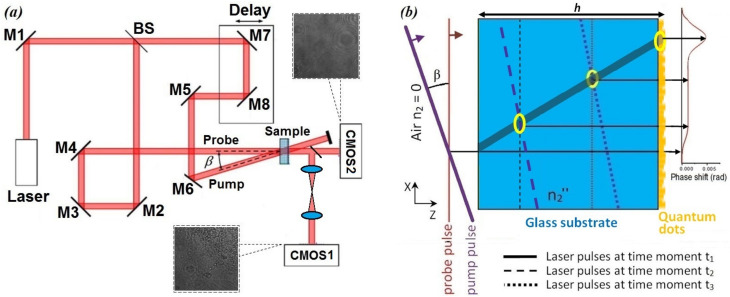
(**a**) A scheme of the experimental setup for the recording of inline digital hologram data set in the TRIDH experiment. (**b**) A scheme of two femtosecond laser pulse propagation inside the sample and probe pulse NDPM-induced phase shift in the XZ plane. Pulses propagation is from the left to right according to the red and purple arrows. The location of both femtosecond pulses in three time moments are denoted with solid, dash, and dotted lines. The plot on the right indicates the amount of phase shift induced in the probe pulse at each point of the X-axis. The gray semitransparent area indicates the pulse intersection area, and the blue and orange indicate the glass substrate and deposited QDs.

Our method was based on the analysis of inline digital holograms of the probe pulse, which propagates inside the studied sample along with a femtosecond pump pulse with the same wavelength but much higher energy density. The optical setup (Figure 1a) utilizes a Ti: Sapphire laser system (regenerative amplifier Regulus 35F1K, Avesta Project Ltd., Moscow, Russia, 2.3 mJ pulse energy, 35 fs pulse duration, 800 nm central wavelength, 30 nm spectral width, and 1 kHz repetition rate), in which the beam is split into the pump and probe beams, 96% and 2% of total energy, respectively. These beams cross each other in the horizontal plane under an adjustable acute angle $\beta \approx 30^{{}^{\circ}}$ and overlapped inside the investigated sample S. The incident angle of $\beta \approx 30^{{}^{\circ}}$ allowed us to efficiently block the scattered pump pulse and to provide a sufficiently large overlap area [23]. In the case of slightly higher incidence angles, the overlapping area between the pump and probe pulses would be smaller [28]. Due to noncollinear degenerate phase modulation (NDPM) of the probe wave, it diffracts at a moderate distance between the sample and image planes, as demonstrated in Figure 1a. If the desired defocused distance is very small (in our case, ≈2 mm), a 4f lens system can be used for transferring the sample plane closer to the CMOS1 sensor (Mindvision HT-UBS130GM, Shenzhen Mindvision Technology Co., Ltd., Shenzhen, China, 1280 × 960 pixels with size Δx=3.75μm, with infrared filter). A magnification factor of a 4f system M≈2 ensured the formation of an enlarged image with an effective pixel $\hat{\Delta x}=\Delta x/M.$ Another CMOS2 sensor (FLIR BFLY-u3-23s6m-c, Teledyne FLIR LLC., Wilsonville, OR, United States, without infrared filter 1900 × 1200, 5.86 μm) was placed directly behind the sample at a further distance (in our case, ≈15 cm) to record the far-field diffraction pattern. Despite the fact that a radiation wavelength of 800 nm is outside the visible range, some cameras are quite sensitive to it, especially if the infrared filter is removed. In our case, we used cameras of both types: without and with an infrared filter. In the latter case, the exposure time was increased to ensure a sufficient signal-to-noise ratio. In this case, the intensity level of the probe beam led to exposure times up to the order of milliseconds. Due to the walk-off effect [29] in the off-axis configuration, the intersection area between the probe and pump pulses is not very large and is slightly shifted during their propagation inside the sample (see the semitransparent gray area in Figure 1b).

Due to rapid relaxation of the refractive index gradient induced by the presence of the pump pulse, deformation of the probe wave and accumulation of the NDPM-induced phase shift takes place only within a local area, where the two pulses actually exist at the same time. Propagation of the probe pulse from the exit plane of the sample to the image plane results in the generation of a specific diffraction pattern, which carries information about the probe pulse phase shift induced during NDPM. Variation in the pump pulse delay line results in a shift in the intersection area between the two pulses, and thus, the sample can be scanned along the X-axis. In order to increase the image quality, each inline hologram was averaged over 20 frames. This allowed us to reduce the impact of shot noise and vibrations as well as to increase the signal/noise ratio by about four times. Experimental data acquisition included the recording of two sets of inline digital holograms by means of two CMOS sensors with sample scanning using the pump pulse delay line. Note that, for correct data processing, it is important to provide low vibrations level, high sample stability, and constant exposure time from the CMOS sensors as well as constant power density from the pump and probe beams.

## 4. Analysis of Probe Pulse Inline Holograms Due to NDPM Inside the Glass Substrate

As we noted above, variation in the pump pulse optical path length results in a shift in the intersection region of the probe and pump pulses inside the sample under study. Therefore, a shift in the pump pulse delay line leads to displacement of the probe beam diffraction pattern, induced by the phase shift introduced during noncollinear degenerate phase modulation inside the sample with a nonlinear refractive index $n_{2}\left(x,y,z\right)$. The simplest case of such an NDPM-induced diffraction can be observed when the major optical characteristics (including nonlinear refractive index) are uniform within the considered area of the sample. In this case, as we described in [23], the diffraction pattern extracted from probe beam is a set of straight vertical interference fringes. In general, such a diffraction pattern resembles the diffraction of a coherent plane wave on a slit.

**Figure 2:**
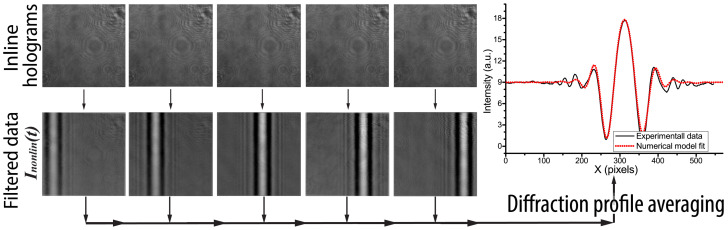
Examples of initial inline digital holograms before (**top** row) and after (**bottom** row) image filtration. The plot on the right side demonstrates the experimental profile and its fitting using numerically simulated data.

A similar pattern was also observed in the far-field diffraction zone when analyzing the area of the studied sample without deposited QDs (in this case, a sample glass with a thickness of 1 mm made of SLIB-G10-050 glass, Labbox Labware, S.L., Barcelona, Spain) (Figure 2). The figure demonstrates a set of diffraction patterns corresponding to different values of the pump pulse delay line, which results in a horizontal displacement of the diffraction pattern clearly visible in the bottom row in Figure 2. For better visualization and data processing, we subtracted the “background” intensity distribution, which does not depend on the pump pulse delay line. Visualization 1 in Supplementary Materials demonstrates a set of initial inline digital holograms corresponding to the movement of the pump pulse delay line.

A numerical analysis of the set of time-resolved inline digital holograms obtained and described in our previous work [23] allowed us to obtain the averaged diffraction profile and to fit it using the numerical model (see the right inset in Figure 2). Data processing of the digital hologram set obtained briefly consists of two major steps: averaging over several consequently recorded digital holograms (in our case, 20 images) and subtracting the background intensity distribution corresponding to the absence of the pump pulse. The whole data set processing (consisting of 30 holograms) typically takes less than 1 min. The data processing allowed us to evaluate the absolute value of the nonlinear refractive index of the sample glass as $|n_{2}^{glass}|\approx 6.3\;\times \;10^{-16}$ cm${}^{2}$/W, which is in good agreement with the $n_{2}$ values published in the literature [23,30].

## 5. Analysis of Probe Pulse Diffraction Due to NDPM on the QDs Deposited onto the Sample Glass

For further investigation of the nonlinear optical properties of the studied sample, we utilized the set of inline digital holograms recorded using the CMOS sensor with a small diffraction distance. It allowed us to obtain the robust diffraction patterns of the probe pulse NDPM on the QDs, deposited onto the glass substrate. In this part of the study, the glass was fixed in such a way that the upper part of the imaged sample contained a QD layer whereas the lower part of the sample was a pure glass substrate, though a small amount of QDs were actually deposited there by accident.

### 5.1. General Type of Inline Digital Hologram Observed in the Experiment

In our previous work [28], we discussed two cases of inhomogeneous distributions of the nonlinear refractive index and analysis of the data obtained for evaluation of the nonlinear optical properties of the sample under study. In particular, we considered the cases of a multilayer medium as well as a sample consisting of a homogeneous substrate and deposited rare small particles, representing local inhomogeneities in the nonlinear refractive index.

In the case of a relatively low concentration of such local inhomogeneities, when a set of inline digital holograms is recorded in the near-field zone, individual local inhomogeneities of the nonlinear refractive index can be distinguished from each other and their diffraction patterns can be analyzed separately. In this case, it is possible to consider the diffraction profiles induced by NDPM on each individual “particle” and to evaluate the nonlinear optical properties of the inhomogeneities by estimating the diffraction pattern modulation amplitude and its asymmetry (see Sections 4B1 and 4B2 in [28]). Such a set of inline digital holograms was obtained recently when analyzing a sample glass with rare graphene particles deposited onto it. Examples of such inline digital holograms were presented in Figure 1c in [31] and will be considered in detail in our future work. In this case, for the numerical simulation of a probe pulse NDPM on such a local nonlinear refractive index inhomogeneity [28], it is possible to find appropriate experimental parameters and to fit the experimentally recorded diffraction profile with a numerically simulated diffraction curve, as demonstrated in Figure 1d of our previous work [31].

**Figure 3:**
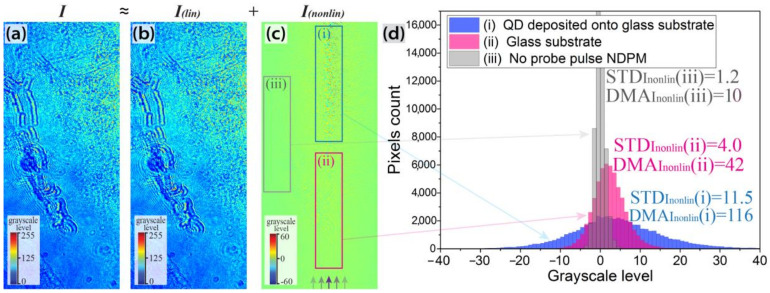
Examples of (**a**) an inline digital holograms recorded at the delay time $t_{1}$, (**b**) a “basic” diffraction pattern $I_{lin}$ as observed without pump pulse, and (**c**) the diffraction pattern of the probe pulse $I_{nonlin}$ induced by its NDPM on the pump pulse in the area of their intersection inside the sample (purple arrows indicate the area of two pulses intersection). Notice the different legend ranges in the figures. (**d**) Examples of grayscale level histograms within the three areas of the $I_{nonlin}$ diffraction patterns of (i) QDs deposited on the glass substrate, (ii) the pure sample glass only, and (iii) no intersection between pump and pulses inside the sample. Standard deviations of the histograms $STD_{I_{nonlin}}$ and diffraction pattern modulation amplitude DMA are indicated for the three areas.

However, in the case of a high concentration of nonlinear refractive index inhomogeneities, the diffraction pattern induced by NDPM of the probe pulse inside the sample is a rather chaotic intensity distribution, where no individual local inhomogeneities of nonlinear refractive index can be distinguished. In this study, due to the high concentration of QDs deposited onto the glass substrate (there were actually several overlapping QD layers), we observed this type of probe pulse inline hologram (see Figure 3a).

### 5.2. Evaluation of Absolute Nonlinear Refractive Index of QDs Deposited onto the Glass Substrate

Due to the differences between the refractive and absorption indexes of the QDs and the environment, some diffraction in the probe wave was observed even in the absence of a pump pulse (Figure 3b). However, when the pump pulse is present, the recorded inline digital hologram (Figure 3a) $I\approx I_{lin}+I_{nonlin}$ consists of two diffraction patterns: 1 is the “basic” diffraction pattern $I_{lin}$ induced by probe wave diffraction on the inhomogeneities of linear optical properties, i.e., absorption and refractive indexes of the sample (Figure 3b); 2 is the diffraction pattern of the probe wave $I_{nonlin}$ due to NDPM by the pump pulse in the area of their intersection inside the sample (Figure 3c). In this case, the diffraction caused by noncollinear degenerate phase modulation appears to be a moving local distortion of the “basic” diffraction pattern when varying the pump pulse delay line. An example of such a set of diffraction patterns can be found in Visualization 2 in Supplementary Materials.

In this case, due to the complex arrangement of the QDs on the sample glass surface, it is not possible to accurately simulate the probe pulse NDPM and diffraction pattern. However, a rough estimation of nonlinear optical properties of the sample can be performed by analyzing the diffraction pattern induced by NDPM $I_{nonlin}$ after its separation from the “basic” diffraction resulting from inhomogeneity of linear optical properties. In order to do that, the “basic” diffraction pattern $I_{lin}$, recorded in the absence of the pump pulse, should be subtracted from each of the inline digital holograms sets. The obtained set of intensity distributions can be further filtered from shot and coherent noise if required. An example of the final pattern $I_{nonlin}$ is demonstrated in Figure 3c. The purple arrows indicate the area where intersection of the probe and pump pulses inside the sample took place at a given delay $t_{1}$.

Figure 3c clearly shows a chaotic diffraction pattern in the upper part of the sample, where QDs have been deposited onto the sample glass. It should be noted that the diffraction pattern in the upper part of the image is due to the probe pulse NDPM inside both the sample glass substrate and the QD layer. Due to the chaotic structure of the probe pulse diffraction on a QD layer, an accurate analysis of such a diffraction pattern is not possible. However, its nonlinear properties can be roughly estimated by evaluating the typical diffraction pattern modulation amplitude (DMA) within the area as a difference between the maximum and minimum intensity $DMA_{I_{nonlin}}\approx \max\left(I_{nonlin}\right)-\min\left(I_{nonlin}\right)$ and its further comparison with the diffraction response of a clean substrate in the lower part of the sample. Taking into account the fact that the diffraction pattern modulation amplitude is roughly proportional to the induced probe wave phase shift (see Section 4B1 in [28]), the absolute value of nonlinear refractive index can be estimated assuming an a priori known thickness of the layer:

(1) $$DMA∼\varphi_{nonlin}\approx P\frac{2\pi }{\lambda }\int_{0}^{h}\left|n_{2}\left(z\right)\right|dz,$$

where *P* is the power density of the pump pulse and *h* is the total thickness of the object. In order to do this, one can evaluate the $DMA_{QD}$ value inside a local rectangular area and compare it with the $DMA_{ref}$ value detected in the ‘reference’ area, corresponding to the pure glass substrate with a previously found nonlinear refractive index of $n_{2}^{glass}$. However, both maximum and minimum intensity values within these rectangular local areas (see examples highlighted in blue and red in Figure 3c) are subject to thet high impact of shot noise, so to increase the robustness of the approach, we suggest analyzing the whole statistical distribution of the intensity values within the area and evaluating its standard deviation, which is less susceptible to random errors:

(2) $$STD_{I_{nonlin}}\left(x,y\right)=\sqrt{\frac{\sum_{i\in [x-l,x+l]\cap [y-l:y+l]}^{n}{\left[I_{nonlin}\left(i\right)-\bar{I_{nonlin}}\right]}^{2}}{n-1}}$$

The examples of the histograms and the result of $STD_{I_{nonlin}}$ evaluation in three large areas of the $I_{nonlin}$ are presented in Figure 3d. The three areas correspond to the regions with (i) QDs deposited on the glass substrate, (ii) a pure sample glass only (although a small amount of QDs was deposited there by accident), and (iii) no intersection between the pump and probe pulses inside the sample. Note that the minor variation of 1.2 in $I_{nonlin}$ in the third area is due to shot and coherent noise, although it is significantly smaller than $STD_{I_{nonlin}}\left(i\right)=11.5$ and $STD_{I_{nonlin}}\left(ii\right)=4.0$ in the first and second areas. Since diffraction within the first area is due to both NDPM in the glass substrate and QDs and since $STD_{I_{nonlin}}\left(ii\right)$ corresponds to the NDPM in a glass substrate only, the phase shift induced by the QD layer is $\left[STD_{I_{nonlin}}\left(i\right)-STD_{I_{nonlin}}\left(ii\right)\right]/STD_{I_{nonlin}}\left(ii\right)\approx 1.88$ times higher than that of the glass substrate ($\varphi_{n_{2}^{QD}}$/$\varphi_{n_{2}^{glass}}\approx 1.88$). Taking into account the nonlinear refractive index of the glass substrate $|n_{2}^{glass}|\approx 6.3\;\times \;10^{-16}$ cm${}^{2}$/W, its thickness $h^{glass}=1$ mm, and the thickness of QDs layer deposited onto the sample glass $h^{QDlayer}=200$ nm, the nonlinear refractive index of the QD layer can be evaluated as follows:

(3) $$|n_{2}^{QDlayer}|=\frac{STD_{I_{nonlin}}-STD_{I_{lin}}}{STD_{I_{lin}}}\cdot \frac{|n_{2}^{glass}|\cdot h^{glass}}{h^{QDlayer}}.$$

The value of $|n_{2}^{QDlayer}|\approx 5.9\;\times \;10^{-12}$ cm${}^{2}$/W obtained agrees with the data on the nonlinear optical properties of similar CdSe/ZnS QDs reported in the literature [13,14,15,16,32].

## 6. Evaluation of the Optical “Nonlinearity Map”

Besides the estimates of the general nonlinear refractive index of QDs, one can evaluate the spatial distribution representing the optical nonlinearity of a sample. In order to do so, diffraction modulation amplitudes of multiple small areas around each pixel should be analyzed (e.g., rectangular or circular area with an area of 100–500 pixels). In our case, a circular pattern with the area of 316 pixels (10 pixels radius) was used by considering intensity histograms within the areas and by calculating $STD_{I_{nonlin}}$ values as performed in Section 5.2. Note that larger analyzed areas result in a decrease in the spatial resolution, although more processed pixels enhance the accuracy of the method. When an estimation of relative optical nonlinearity of several large areas with deposited nanoparticles within the field of view is required, the area size can be increased up to 10,000 or even more pixels. However, sample monitoring with high spatial resolution and reasonable accuracy can be achieved by analyzing small areas with only 100–150 pixels, although we do not recommend using areas with less than 100 pixels due to the strong shot noise impact.

**Figure 4:**
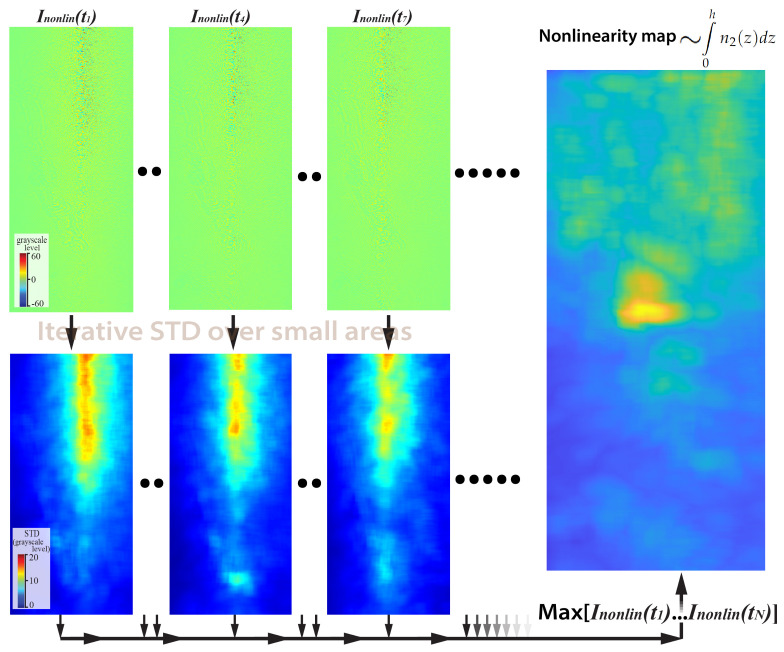
The top row demonstrates diffraction patterns induced by the femtosecond probe pulse NDPM. The bottom row shows several typical nonlinearity “maps” obtained for different pump pulse delay values. The final nonlinearity “map”, estimated from their combined analysis, is shown in the right part of the figure.

When such a “map” is calculated for a single inline digital hologram, the data on only a small portion of the sample area are obtained. The area is limited by the intersecting region between the pump and probe pulse. In order to extend the information about the sample, the process should be repeated for other inline digital holograms with shifted intersection regions (see the left part of Figure 4). Finally, the images obtained should be combined and a single “nonlinearity map” can be constructed. In fact, such an image represents an integral amount of the nonlinear refractive index of the sample $\int_{0}^{h}\left|n_{2}\left(z\right)\right|dz$, which affects the probe pulse when it propagates across the sample.

In the case of our experimental data processing, several inline digital holograms data sets were recorded with slightly varying defocusing distances within the range of 1–3 mm. Each of the sets was processed according to the abovementioned protocol, and afterward, the nonlinearity maps obtained were averaged. The final nonlinearity map of the sample is presented in Figure 4. The resultant figure clearly shows a significant difference in estimated nonlinearity within the top and bottom parts of the sample. This result agrees well with the sample preparation procedure, assuming only partial QD deposition.

## 7. Discussion

In this paper, for the first time, we presented the experimental implementation of time-resolved inline digital holography for the analysis of nonlinear optical properties of a sample glass with a deposited QD layer. Several sets of inline digital holograms were recorded according to the TRIDH approach on the pure glass substrate area of the sample and within the region where QDs were deposited onto the surface. An analysis of the inline digital holograms obtained along with a priori knowledge of the sample glass thickness and QD layer thickness allowed us to evaluate their nonlinear refractive indexes. The results obtained for the BK-7 glass $|n_{2}\left(glass\right)|$ = $6.3\;\times \;10^{-16}$ cm${}^{2}$/W and QDs $|n_{2}\left(QD\right)|=5.9\;\times \;10^{-12}$ cm${}^{2}$/W agree well with typical data presented in the literature for BK-7 glass and similar QDs, respectively. Taking into account that the QD layer thickness could be nonuniform across the sample and that nonlinear refractive indexes of QDs may not be constant and may depend, e.g., on their size varying within a certain range [13,14,15,16,33], we evaluated two-dimensional nonlinearity maps within a small sample area. The final “nonlinearity map” represents the integral amount of phase shift into the probe wavefront propagating across the sample. The “nonlinearity map” obtained (see Figure 4) allowed us to clearly distinguish between the areas, where QDs were actually deposited onto the sample glass (upper part of the image) and where no QDs were present (lower part of the image).

The possibilities of the proposed technique can be expanded with the involvement of nondegenerate phase modulation when the pump and probe radiation differ in wavelength. The use of parametric generators of femtosecond radiation will make it possible to study the spectrally dependent nonlinear properties of the samples [34,35] in addition to the utilized spatial degree of freedom.

## Data Availability

The data are contained within the article and in the Supplementary Materials.

## Supplementary Materials

The following supporting information can be downloaded at: https://www.mdpi.com/article/10.3390/jimaging8030074/s1, Visualization 1 demonstrates a set of inline digital holograms captured by CMOS2 in a far diffraction zone during the movement of the pump pulse delay line. Visualization 2 demonstrates a set of inline digital holograms captured by CMOS1 in the near diffraction zone during the movement of the pump pulse delay line.
