# Supplementary figures and images for: Investigation of Nonlinear Optical Properties of Quantum Dots Deposited onto a Sample Glass Using Time-Resolved Inline Digital Holography
Source: J Imaging. 2022 Mar 16;8(3):74. doi: 10.3390/jimaging8030074 (PMC8955446; doi:10.3390/jimaging8030074)

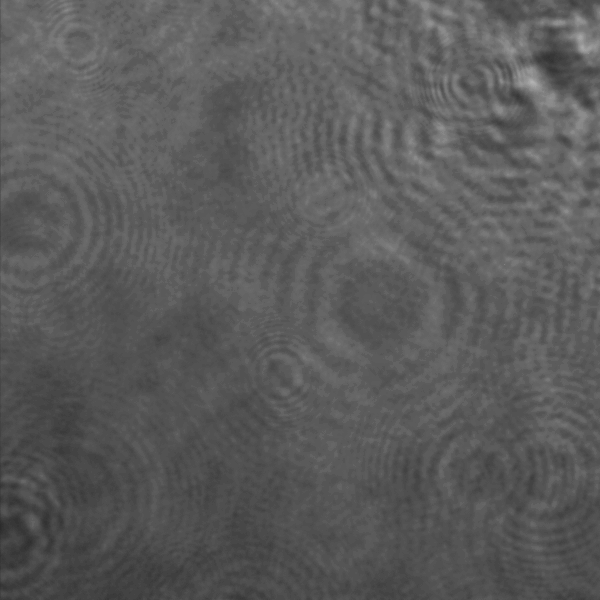

Supplement: Supplementary file 1 [file jimaging-08-00074-s001.zip › Visualization1.gif]

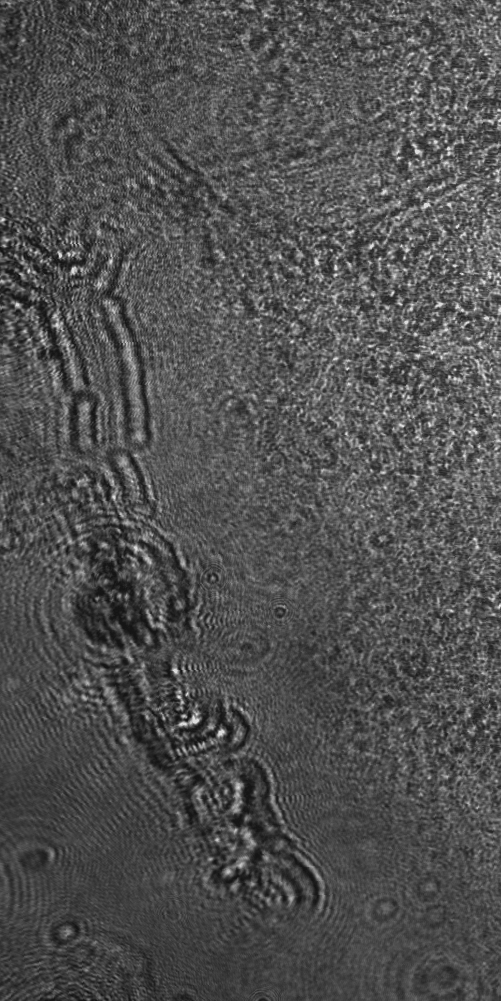

Supplement: Supplementary file 1 [file jimaging-08-00074-s001.zip › Visualization2.gif]
